# The de novo, chromosome-level genome assembly of the sweet chestnut (*Castanea sativa* Mill.) Cv. Marrone Di Chiusa Pesio

**DOI:** 10.1186/s12863-024-01245-7

**Published:** 2024-06-22

**Authors:** Luca Bianco, Paolo Fontana, Alexis Marchesini, Sara Torre, Mirko Moser, Stefano Piazza, Sara Alessandri, Vera Pavese, Paola Pollegioni, Cristiano Vernesi, Mickael Malnoy, Daniela Torello Marinoni, Sergio Murolo, Luca Dondini, Claudia Mattioni, Roberto Botta, Federico Sebastiani, Diego Micheletti, Luisa Palmieri

**Affiliations:** 1 Research and Innovation Center, Edmund Mach Foundation, via Mach 1, San Michele all’Adige, TN 38098 Italy; 2 Research Institute on Terrestrial Ecosystem, National Research Council, Via Marconi 2, Porano, TR 05010 Italy; 3 NBFC, National Biodiversity Future Center, Palermo, Italy; 4 Institute for Sustainable Plant Protection, National Research Council, Via Madonna del Piano 10, 50019, Sesto Fiorentino FI, Italy; 5 Dept. of Agricultural and Food Sciences, University of Bologna, Via Zamboni 33, Bologna, BO 40126 Italy; 6 Dept. of Agricultural, Forest and Food Sci, University of Turin, L.go P. Braccini 2, Grugliasco, TO 10095 Italy; 7 Dep. of Agricultural, Food and Env.Sci, Marche Polytechnic University, via Brecce Bianche, Ancona, AN 60131 Italy

**Keywords:** European chestnut, Genome sequence assembly, Chromosome-scale

## Abstract

**Objectives:**

The sweet chestnut *Castanea sativa* Mill. is the only native *Castanea species* in Europe, and it is a tree of high economic value that provides appreciated fruits and valuable wood. In this study, we assembled a high-quality nuclear genome of the ancient Italian chestnut variety ‘Marrone di Chiusa Pesio’ using a combination of Oxford Nanopore Technologies long reads, whole-genome and Omni-C Illumina short reads.

**Data description:**

The genome was assembled into 238 scaffolds with an N50 size of 21.8 Mb and an N80 size of 7.1 Mb for a total assembled sequence of 750 Mb. The BUSCO assessment revealed that 98.6% of the genome matched the embryophyte dataset, highlighting good completeness of the genetic space. After chromosome-level scaffolding, 12 chromosomes with a total length of 715.8 and 713.0 Mb were constructed for haplotype 1 and haplotype 2, respectively. The repetitive elements represented 37.3% and 37.4% of the total assembled genome in haplotype 1 and haplotype 2, respectively. A total of 57,653 and 58,146 genes were predicted in the two haplotypes, and approximately 73% of the genes were functionally annotated using the EggNOG-mapper. The assembled genome will be a valuable resource and reference for future chestnut breeding and genetic improvement.

**Supplementary Information:**

The online version contains supplementary material available at 10.1186/s12863-024-01245-7.

## Objective

*Castanea* Mill. (2n = 2x = 24) is a genus of broadleaved trees and shrubs of the Fagaceae family that includes seven species (although the taxonomic identity of some entities is still debated [1, 2]) that are native to temperate deciduous forests of the Northern Hemisphere. Among these, three species are cultivated for fruit: Japanese chestnut (*C. crenata* Sieb. et Zucc), Chinese chestnut (*C. mollissima* Bl.), and European chestnut (*C. sativa* Mill.). European chestnut, also known as sweet chestnut, is the only European species of the genus and is native to central-southern Europe (northern Iberian Peninsula, southern France, central-northern Italy, southern Balkan Peninsula) and Asia Minor (western and northern Turkey, Caucasus); however, it has been widely planted and cultivated outside its natural range in temperate regions worldwide (e.g., South and North America and Australia). Sweet chestnut is a species of remarkable ecological and economic importance. In addition to being the dominant tree in its native range, mesophilous, broad-leaved forests have been cultivated for millennia for timber production (coppice and high forest) and fruit production (traditional orchards), providing a broad range of secondary products and ecosystem services [3].

The sweet chestnut Marrone genotype was selected for its exceptional qualities, such as its above-average fruit weight (maximum 70 fruits/kg), mono-embryonic nuts, and thin and easy-to-remove episperm (cuticle), which is not deep in the cotyledons and their floury paste or sugary and is consistent [4, 5]. Reference genomes have been published for three Castanea species so far. In 2020, a scaffold-level genome assembly of *C. mollissima* cultivar N11-1, generated using PacBio sequencing technology, was published (GenBank assembly accession: GCA_014183005.1), and in the same year the scaffold-level genome for *C. mollissima* cultivar “Vanuxem” was assembled [6]. In 2021, contig- and scaffold-level genomes were generated from PacBio sequencing also for *C. crenata* (GenBank: GCA_019972055.1 and GCA_020976635.1). In 2022, a chromosome-level genome assembly was published for *C. crenata* by combining Nanopore long reads and Hi-C sequencing [7]. Two chromosome-scale and haplotype-resolved reference genome assemblies were then recently generated for *C. mollissima*, “Mahogany” and “Nanking” cultivar (HudsonAlpha Institute for Biotechnology; http://phytozome.jgi.doe.gov/info/CmollissimaMahoganyHAP2_v1_1).

Here, we describe a chromosome-scale de novo genome assembly of *Castanea sativa* Mill., cv. “Marrone di Chiusa Pesio”, which, to our knowledge, is the first reported *C. sativa* genome assembly. We believe this study will provide important resources for better investigating the evolutionary history and domestication process of this species and elucidating the genetic basis of resistance to diseases and environmental stressors.

## Data description

Fresh, young leaves were collected from a single true-to-type plant, ‘Marrone di Chiusa Pesio’, which was provided by the Chestnut R&D Center Piemonte (https://centrocastanicoltura.org/en/). DNA extraction was performed using Macherey Nagel’s NucleoSpin Plant II Midi following the manufacturer’s protocol.

Illumina libraries were constructed from the genomic DNA following the Illumina TruSeq kit protocol and sequenced (PE150) by Novogene, yielding 89 Gb of data. Genomic DNA was also sequenced using the ONT Minion device with Flowcell version R9.4.1. Additionally, a Hi-C library was prepared with the Omni-C Kit from Dovetail Genomics following the manufacturer’s protocol with minor adjustments (Supplementary Appendix and Table 1).

K-mer analysis of the Illumina reads with a kmer size of 23 using GenomeScope [8] indicated an estimated genome size of 654 Mbps.

The ONT reads were assembled using the NextDenovo assembler v2.5.0 [9], and the assembled sequence was polished with NextPolish v1.4.0 [10], resulting in 238 scaffolds with an N50 value of 21.8 Mbps and an N80 value of 7.1 Mbps, for a total of 750 Mbps. The scaffold-to-chromosome ratio was 19.83. Chromosome-level scaffolding was performed with Omni-C data with standard parameters (https://omni-c.readthedocs.io/en/latest*).* A manual inspection of the contact maps was conducted without highlighting any issues.

The genome was anchored using two published genetic maps for *Castanea sativa*: ‘Bouche de Betizac’ and ‘Madonna’ [11]. ALLMAPS [12] was used with standard parameters after the SNPs were mapped to the assembled genome and the alignments were filtered (98% identity and coverage of the probe sequence and uniqueness of each haplotype). The marey plots are reported in the Supplementary materials.

The two haplotypes were reconstructed by phasing (with ONT reads) the structural variants identified by Illumina reads with WhatsHap software v.1.0 [13] and the BCFtools v.1.7 consensus command [14].

The final anchored sequence contained 715 Mbps (Haplotype 1) and 713 Mbps (Haplotype 2) (Sup. Table 2).

BUSCO (v. 5.2.2) [15] analysis indicated high genome assembly completeness (98.6%). Furthermore, 97% of the filtered Illumina reads were aligned to the genome assembly using BWA v.0.7.17 [16], with 93% being properly paired (see Supplementary materials).

The LTR assembly index (LAI) was computed with LTR_Retriever v2.9.7 [17] for both haplotypes, with scores of 17.87 for Haplotype 1 and 16.85 for Haplotype 2, indicating good completeness. Repetitive elements in the genome were first identified using EDTA v2.0 [18]; then, RepeatMasker v4.1.2 [19] was used to identify and annotate the repetitive sequences.

Gene prediction was carried out separately for each haplotype. The dataset of mature miRNAs and the corresponding hairpin sequences of Quercus robur, which is the genetically nearest species, were retrieved from the PmiREN data repository [20]. These sequences were independently aligned to the two haplotypes using bowtie [21] (version 1.3.1) for the short miRNA reads and Blastn [22] for the hairpin sequences. The tRNAs were predicted by tRNAscan-SE v2.0.6 [23]. The coding genes prediction was carried out using Augustus v3.4 [24] and Maker v3.01 [25] (GeneMark-ES [26], Augustus and EVidenceModeler v1.1.1 [27]), trained with RNA-Seq data (downloaded from SRA: SRR8305473 and SRR15058346) and proteins from NCBI RefSeq (belonging to the Fagaceae family), respectively. The two predictions were merged according to the results of the GeneValidator v2.1.12 [28] tool, which retained only the best predictions for every gene site.

The genes masked by RepeatMasker were searched for domains associated with resistance genes using hmmsearch [29] and the PFAM domains reported in [30]. The two predictions were merged according to the results of the GeneValidator v2.1.12 tool, which retained only the best predictions for every gene site. The predicted proteins were functionally annotated using EggNOG-mapper [31] and the results were filtered using Fun TaxIS-lite [32].

**Table 1:** Overview of the data files and datasets

Label	Name of data file/dataset	File types (file extension)	Data repository and identifier (DOI or accession number)
*Data file 1*	*Genome Haplotype1*	*Fasta file (.fasta)*	https://treegenesdb.org/FTP/Genomes/.Cast/v1.0/genome/Cast.1_0.hap1.fa [33]
*Data file 2*	*Genome Haplotype2*	*Fasta file (.fasta)*	https://treegenesdb.org/FTP/Genomes/.Cast/v1.0/genome/Cast.1_0.hap2.fa [34]
*Data file 3*	*Gene prediction on Haplotype1*	*GFF3 file (.gff)*	https://treegenesdb.org/FTP/Genomes/.Cast/v1.0/annotation/Cast.1_0.hap1.gff [35]
*Data file 4*	*Gene prediction on Haplotype2*	*GFF3 file (.gff)*	https://treegenesdb.org/FTP/Genomes/.Cast/v1.0/annotation/Cast.1_0.hap2.gff [36]
Data file 5	K-mer spectrum of the Illumina reads	Image file (.png)	10.6084/m9.figshare.25568154 [37]
Data file 6	Omni-C contact maps	Image file (.svg)	10.6084/m9.figshare.25568163 [38]
Data file 7	Busco scores of the assembled sequence	Image file (.png)	10.6084/m9.figshare.25568139 [39]
Data file 8	miRNA analysis	Spreadsheet(.xlsx)	10.6084/m9.figshare.25568064 [40]
Accession identifier	Bioproject identifier	Website (Html)	http://identifiers.org/ncbi/bioproject:PRJNA1096137 [41]
*DataSet 1*	*ONT reads of C. sativa*	*SRA file (.sra)*	http://identifiers.org/ncbi/insdc.sra:SRR28552917 [42]
*DataSet 2*	*Illumina PE-150 reads of C. sativa*	*SRA file (.sra)*	http://identifiers.org/ncbi/insdc.sra:SRR28552918 [43]
*DataSet3*	*Dovetail Omni-C of C. sativa*	*SRA file (.sra)*	http://identifiers.org/ncbi/insdc.sra:SRR28552916 [44]

### Limitations

Although the gene space is quite complete, this assembly lacks resolution of the two haplotypes. The sequences of the two haplotypes were reconstructed based on the phasing of short-read sequencing enhanced with the use of long reads. A better resolution of the two haplotypes might be reached in the future by producing PacBio HiFi reads and integrating them with the data produced in this work.

### Electronic supplementary material

Below is the link to the electronic supplementary material.

Supplementary Material 1

## Data Availability

The data described in this Data note can be freely and openly accessed from the NCBI database under BioProject accession PRJNA1095814 and PRJNA1095812. The sequencing reads are available at the Sequence Read Archive (SRA) under BioProject accession PRJNA1096137. The assembled sequence and gene predictions are available for download at the following address: https://treegenesdb.org/FTP/Genomes/.Cast/v1.0/.

## Abbreviations

ONT: Oxford Nanopore Technologies
SNP: Single Nucleotide Polymorphism
LTR: Long-Terminal Repeat
BUSCO: Benchmarking Universal Single-Copy Orthologs
NCBI: National Center for Biotechnology Information
SRA: Sequence Read Archive
