# Supplementary material for: The de novo, chromosome-level genome assembly of the sweet chestnut (Castanea sativa Mill.) Cv. Marrone Di Chiusa Pesio
Source: BMC Genom Data. 2024 Jun 22;25:64. doi: 10.1186/s12863-024-01245-7 (PMC11193896; doi:10.1186/s12863-024-01245-7)

Appendix

**Library preparation and sequencing.**

Illumina libraries were prepared starting from genomic DNA using the TruSeq kit (illumina, following the manufacturer’s instructions. The libraries were sequenced by Novogene with a PE150 setup and a yield of 89Gb.

Genomic DNA was prepared for sequencing on an Oxford Nanopore Technologies Minion device (<https://nanoporetech.com/products/minion>) using Ligation Sequencing kit (SQK-LSK109) following the manufacturer’s protocol, sequencing was performed using Flowcell version R9.4.1 (FLO-MIN106D; Oxford Nanopore Technologies) for a 72-hours running time of each flow cell used.

The Hi-C library was prepared with the Omni-C kit (Dovetail Genomics, CA, US) according to the manufacturer’s protocol with minor modifications. The nuclei were isolated by grinding the fresh leaves in liquid nitrogen and by filtration with Miracloth followed by centrifugation (details in Workman et al., 2018). Subsequently, chromatin was fixed with formaldehyde, and digested with DNase I until an optimal fragment length distribution of DNA molecules was obtained. The digested DNA was subjected to end polishing, followed by ligation to a biotinylated bridge adapter and proximity ligation of the adapter-containing ends. After proximity ligation, crosslinks were reversed, the DNA purified, and then treated to remove biotin that was not internal to ligated fragments. Biotin-containing fragments were isolated using streptavidin beads and enriched by PCR. The library was sequenced on an Illumina HiSeqX platform to give 2 × 150 bp paired-end reads, producing approximately 30x sequence coverage.

Supplementary Table

Table1: Reads Summary

|  | NCBI SRA | No. of reads | No. of Mbases | % GC |
| --- | --- | --- | --- | --- |
| Illumina PE150 | insdc.sra:SRR28552918 | 297,739,153 | 89,096 | 36.55 |
| ONT | insdc.sra:SRR28552917 | 11,236,518 | 71,547 | 36.67 |
| Dovetail Omni-C | insdc.sra:SRR28552916 | 343,733,110 | 103,120 | 40.08 |

Table 2: Genome Assembly statistics

|  | Haplotype 1 | Haplotype 2 |
| --- | --- | --- |
| Total size | 715,869,006 | 713,038,847 |
| N50 | 59,921,945 | 59,838,338 |
| % GC | 35.41 | 35.40 |
| Repetitive Element (bp) | 266,966,059 | 266,503,914 |
| Genes | 57,653 | 58,146 |

Supplementary Material

1. Marey plots with Bouche de Betizac’s genetic map. The chromosome order is from 1 to 12, corresponding to linkage groups A to L. The physical sequence of the Marrone di Chiusa Pesio is compared against the genetic position of the mapped markers of the Bouche de Betizac’s genetic map.


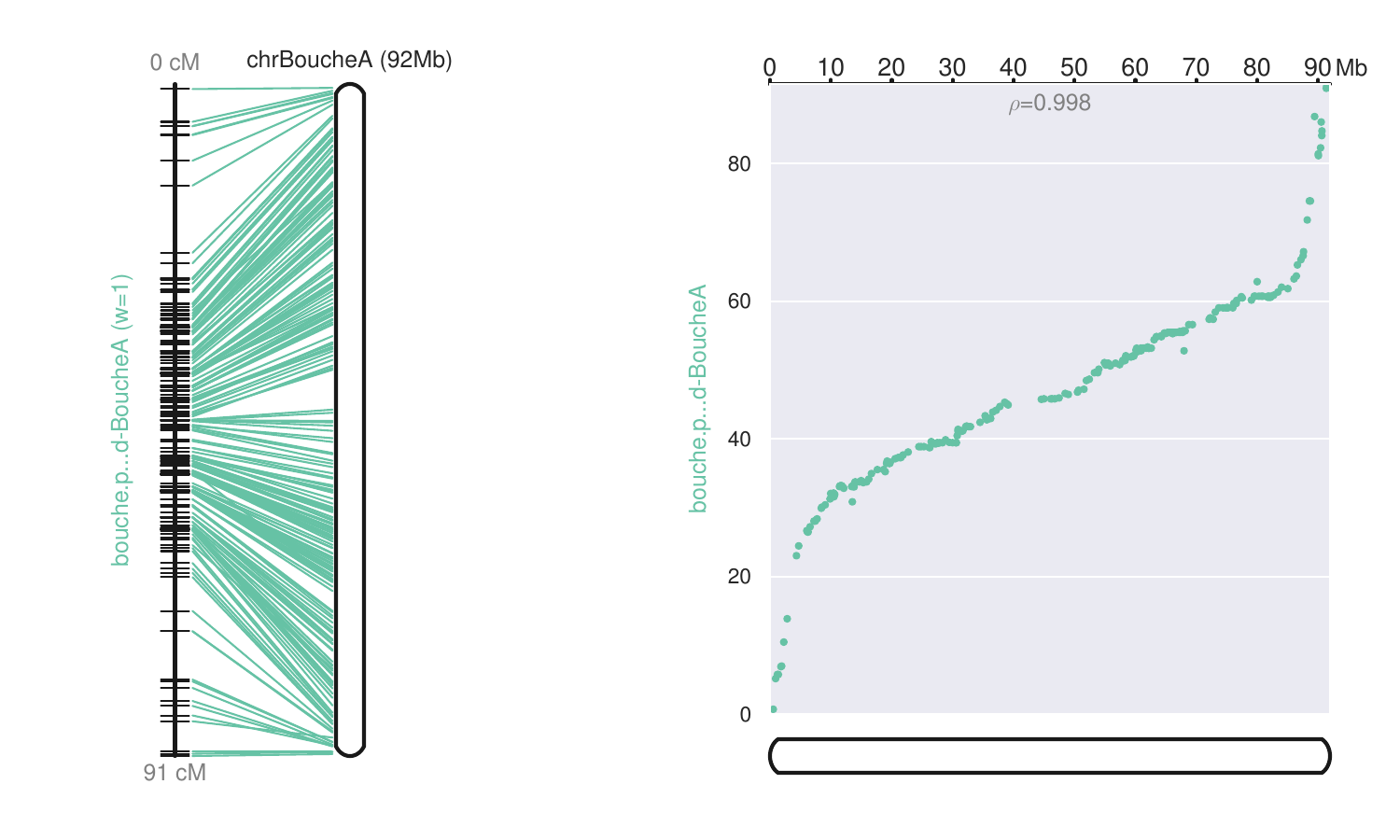

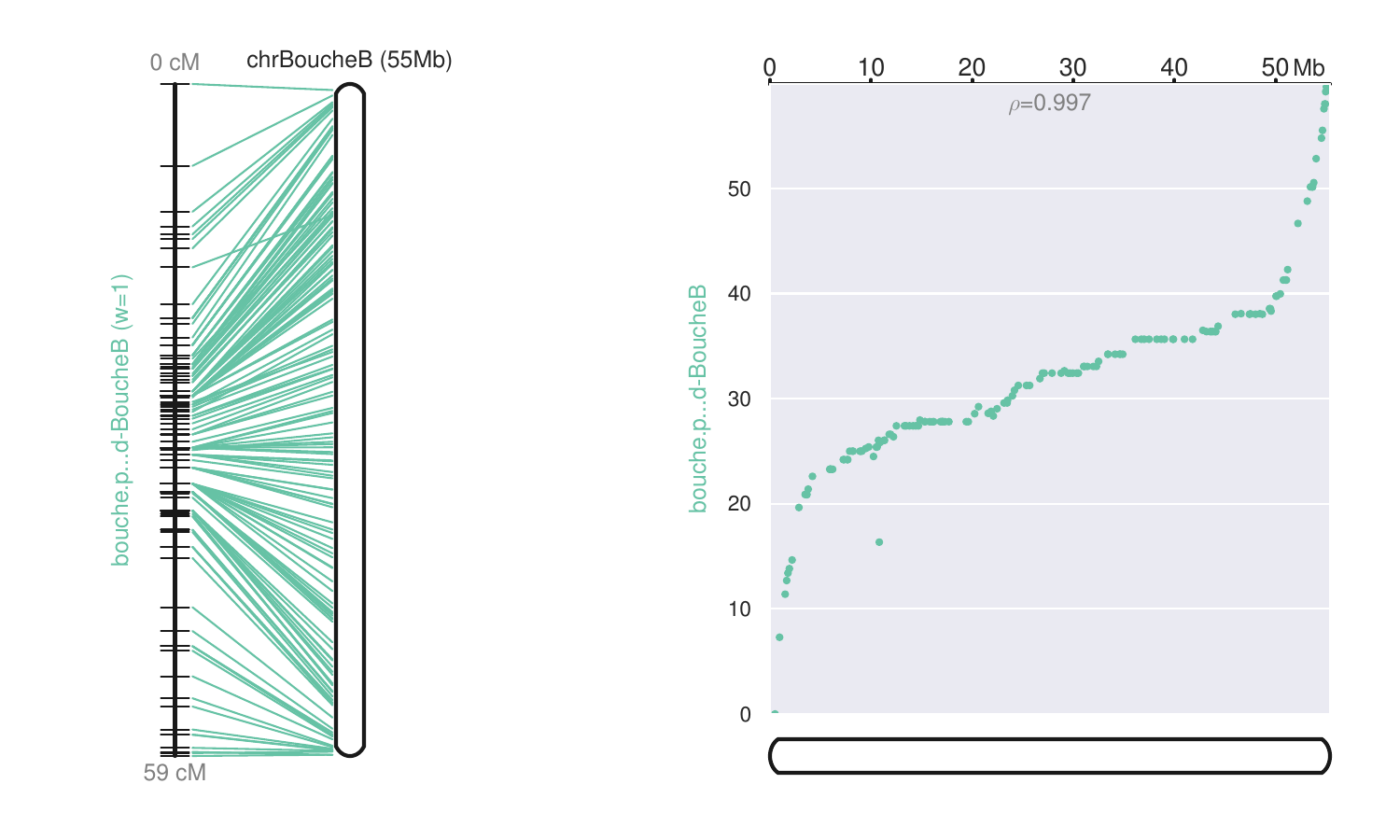

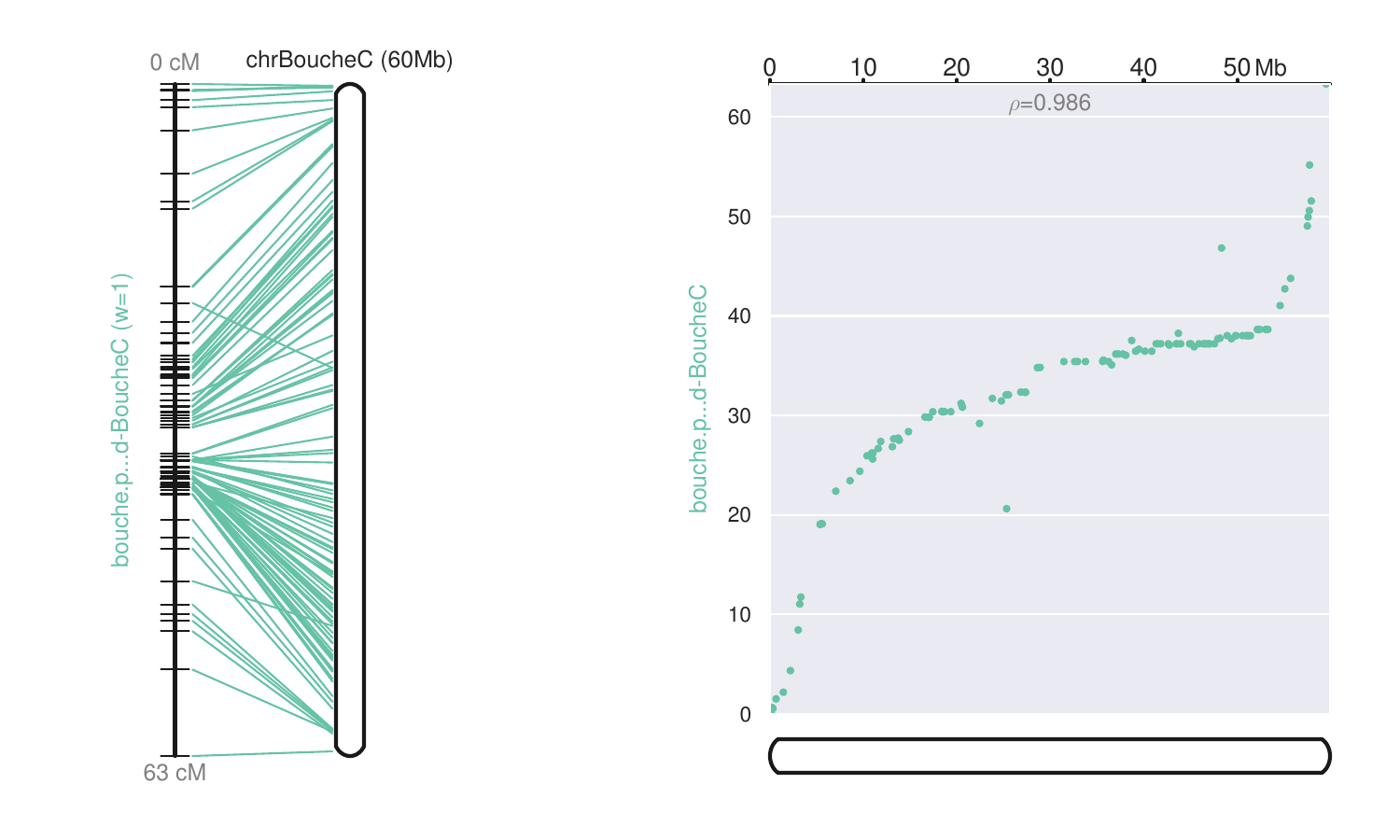

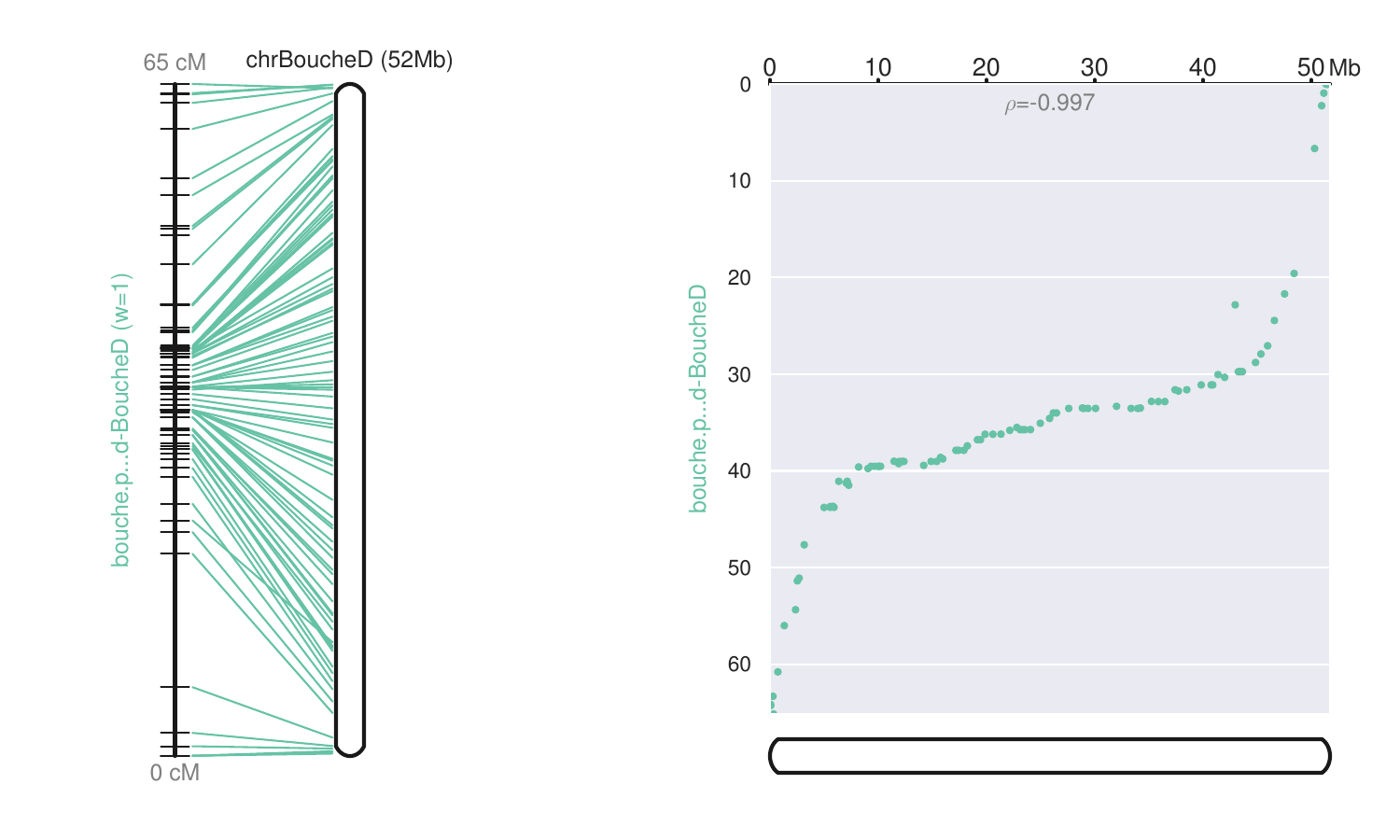

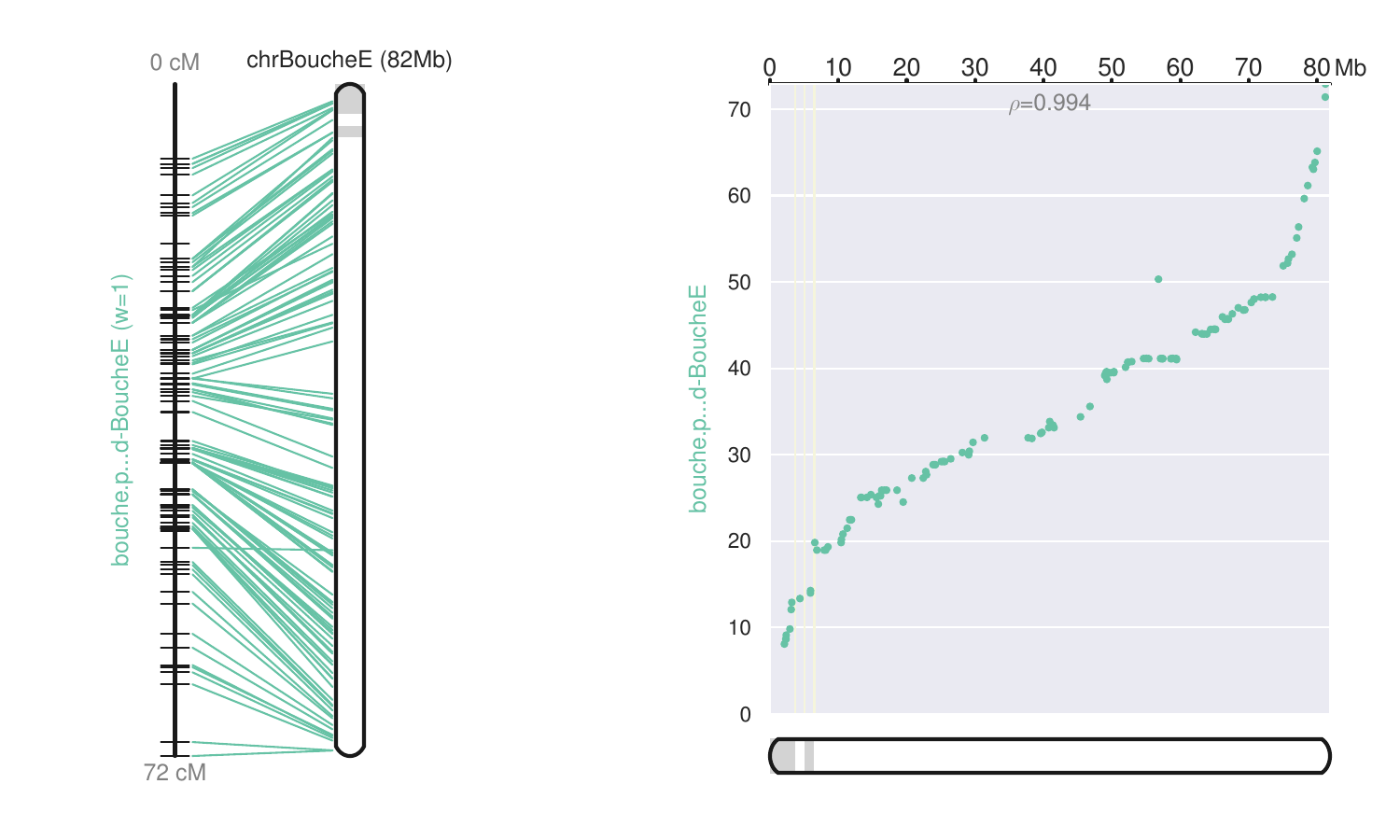

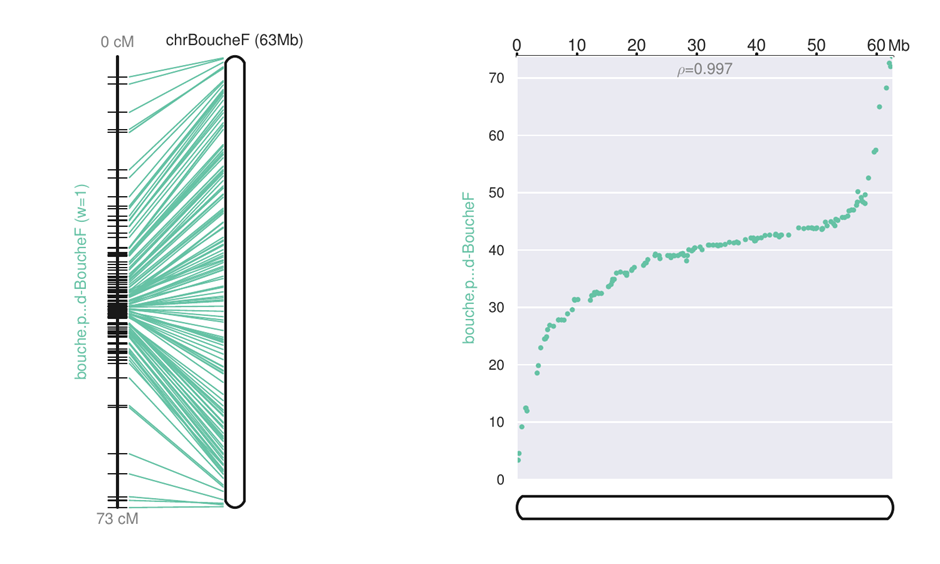


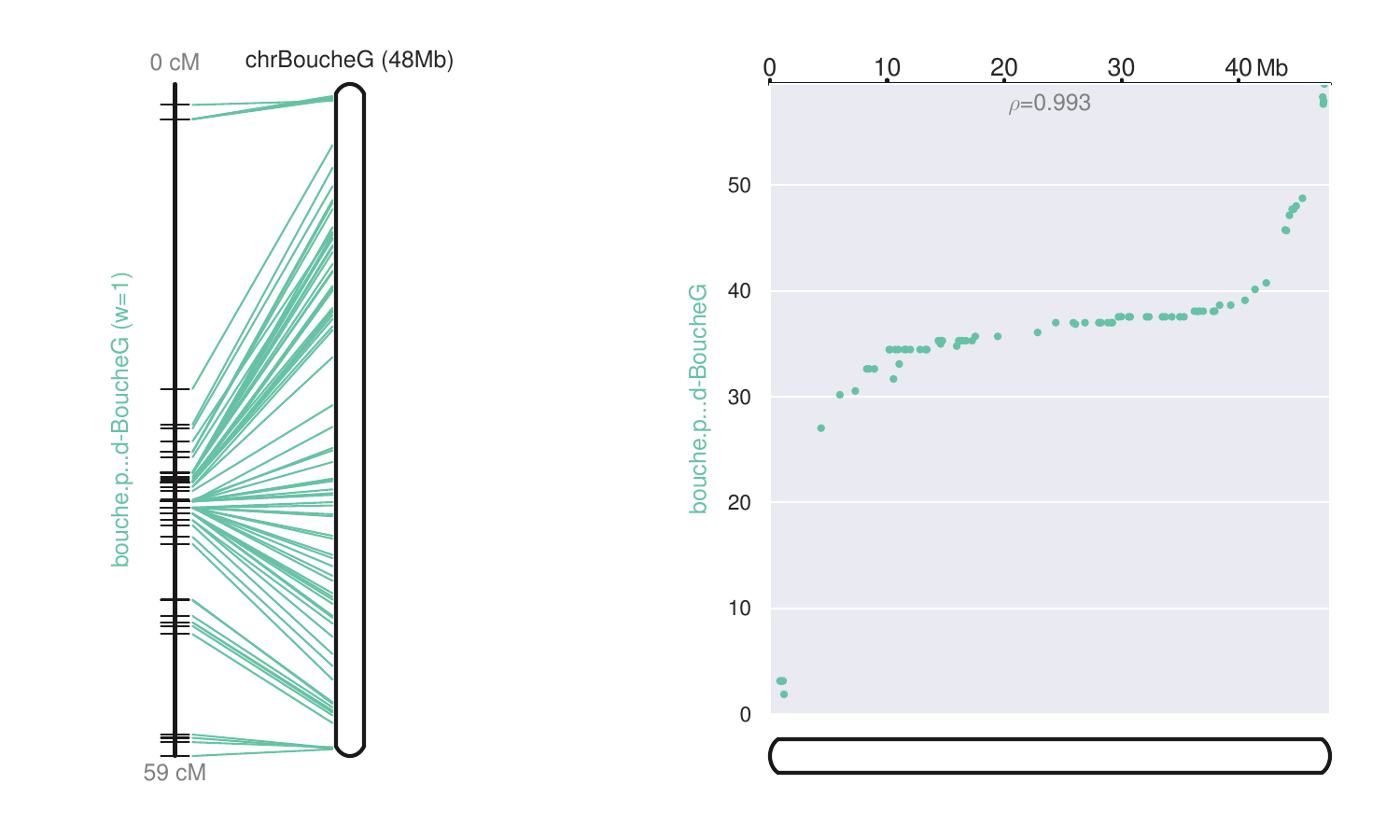

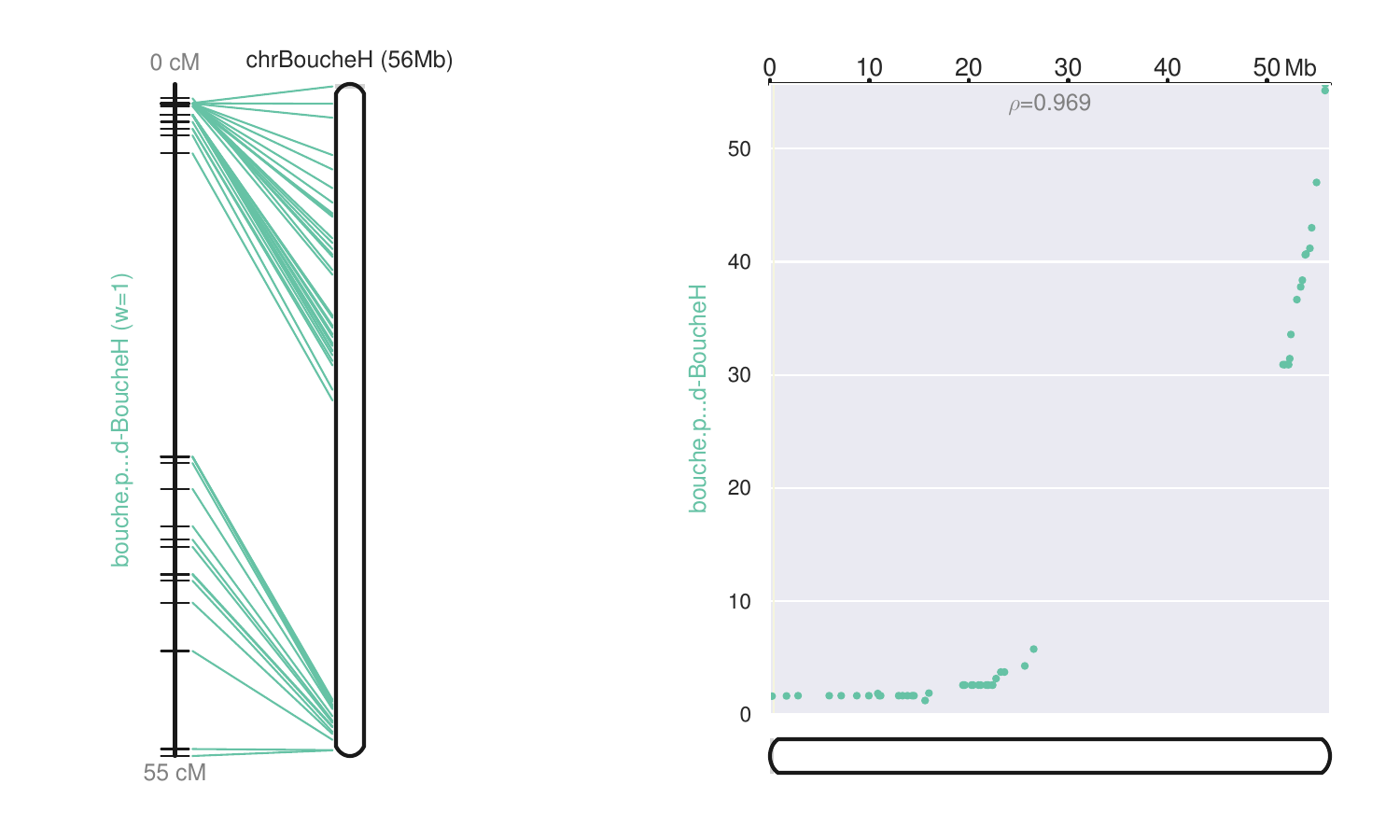

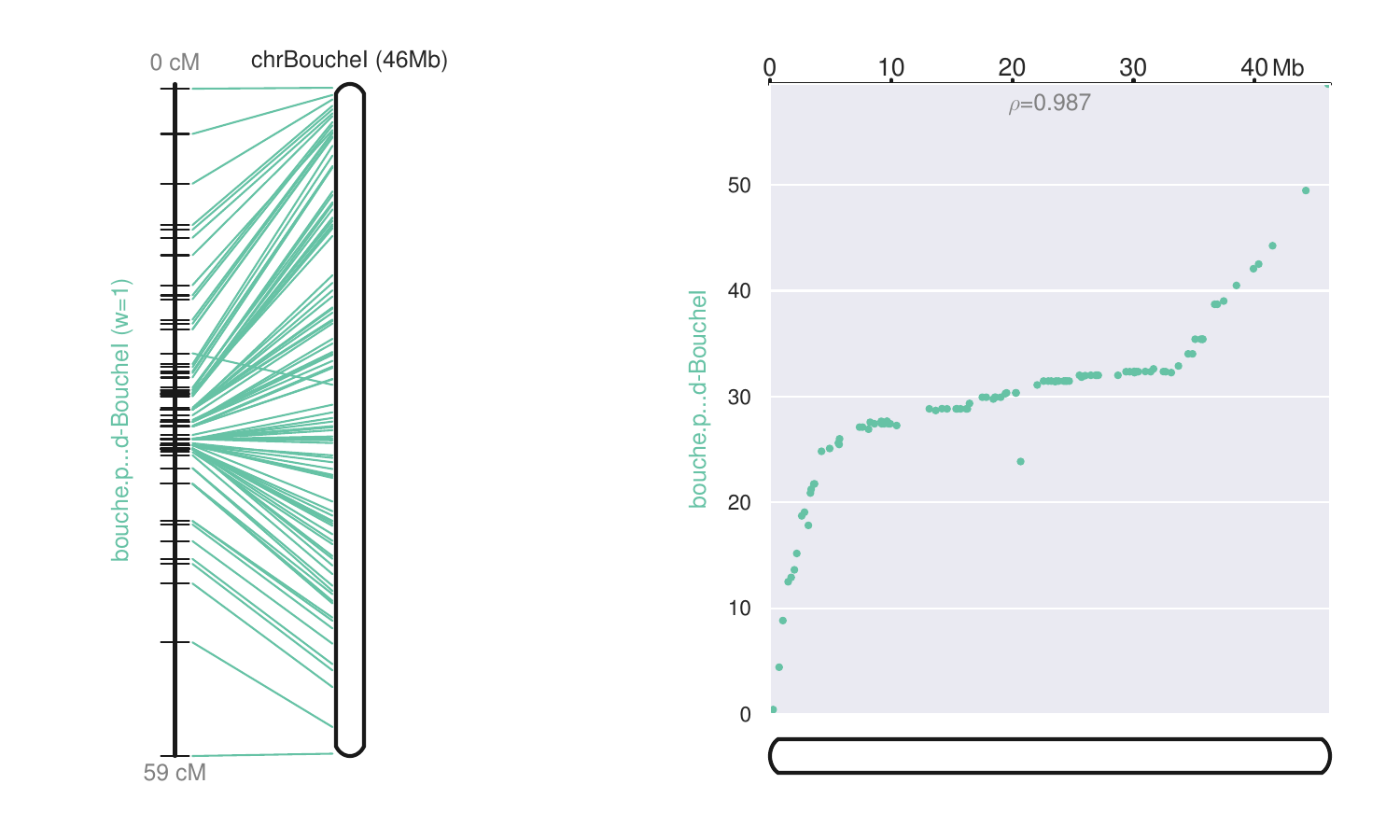

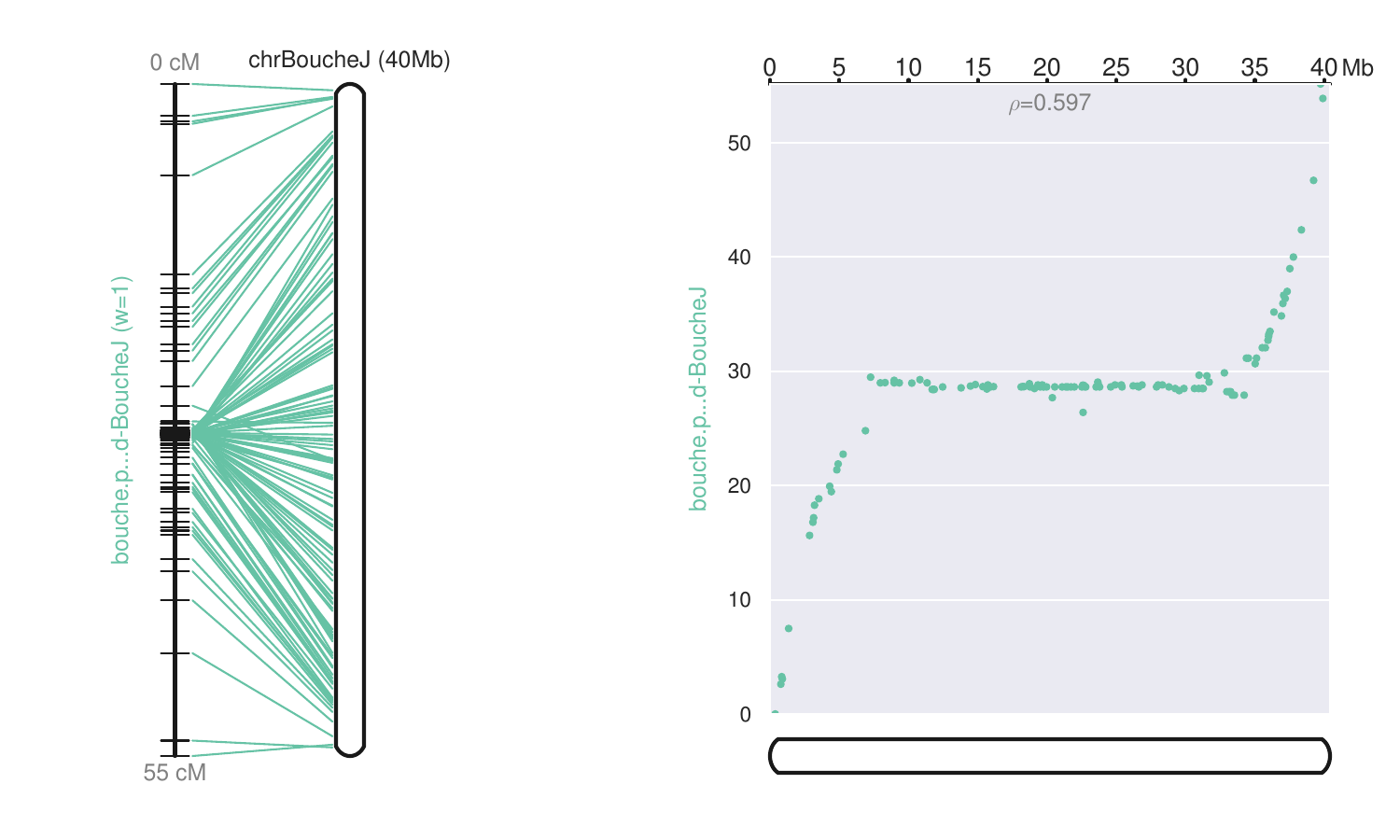

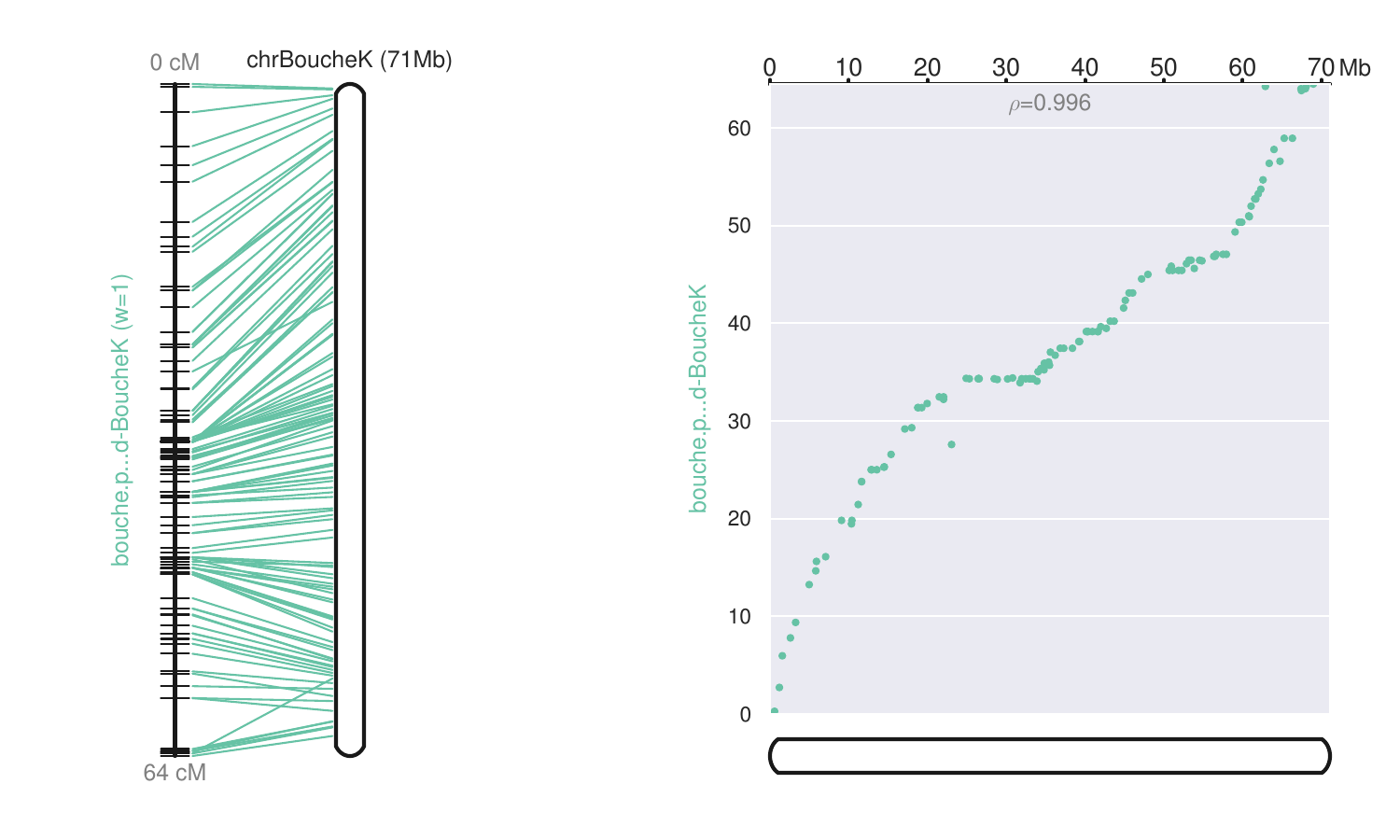

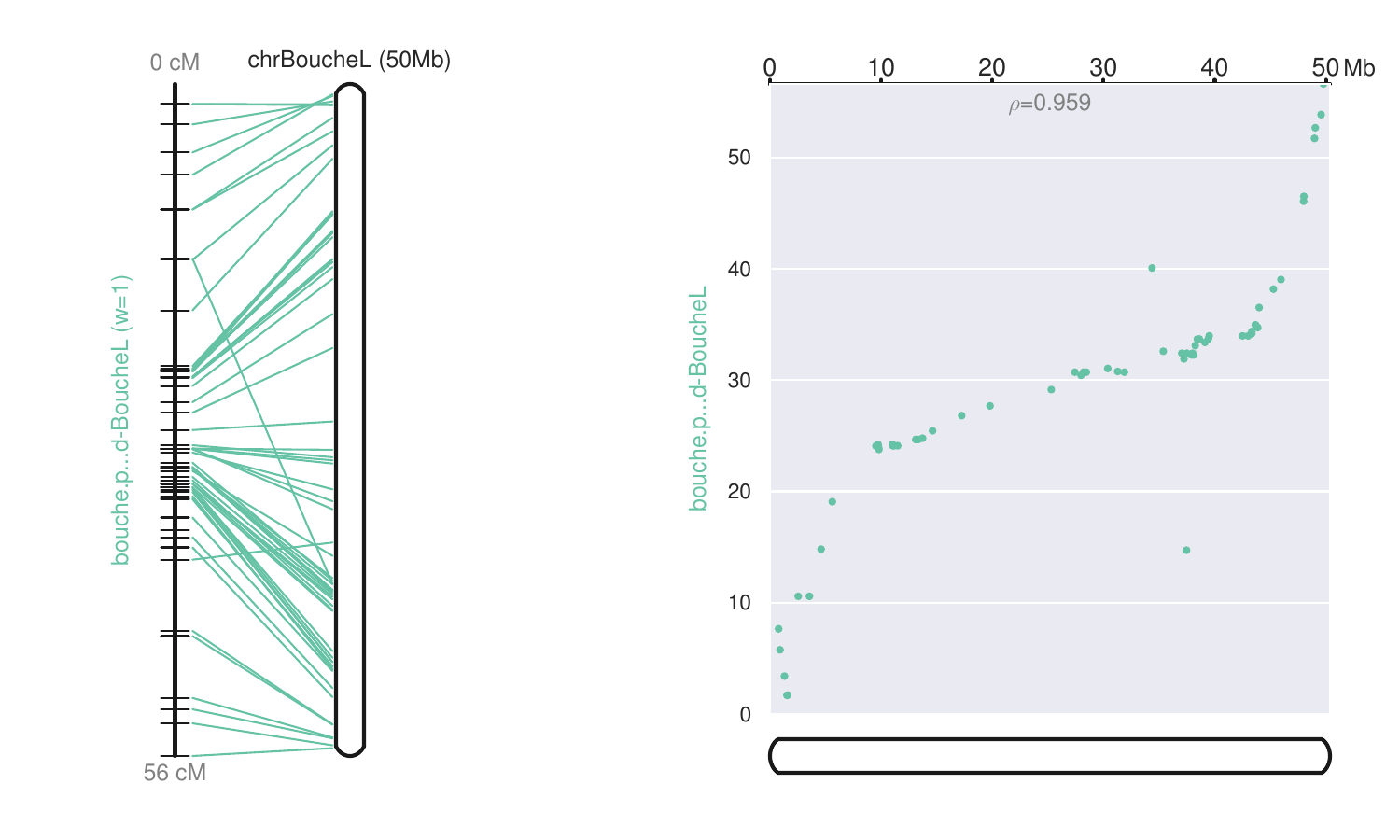


1. Marey plots with Madonna’s genetic map (chromosome order is from 1 to 12). The chromosome order is from 1 to 12, corresponding to linkage groups A to L. The physical sequence of the Marrone di Chiusa-Pesio is compared against the genetic position of the mapped markers of the Madonna’s genetic map.


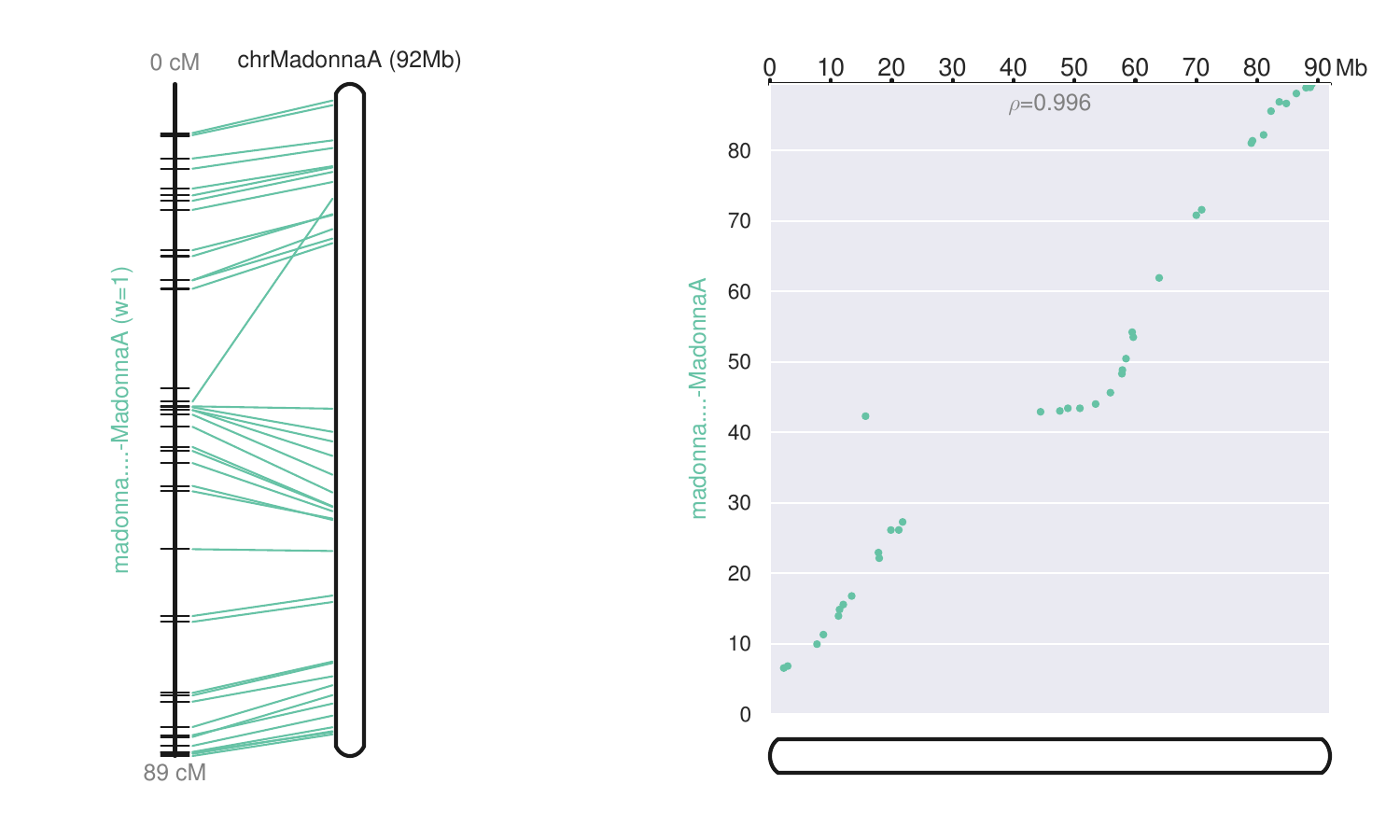

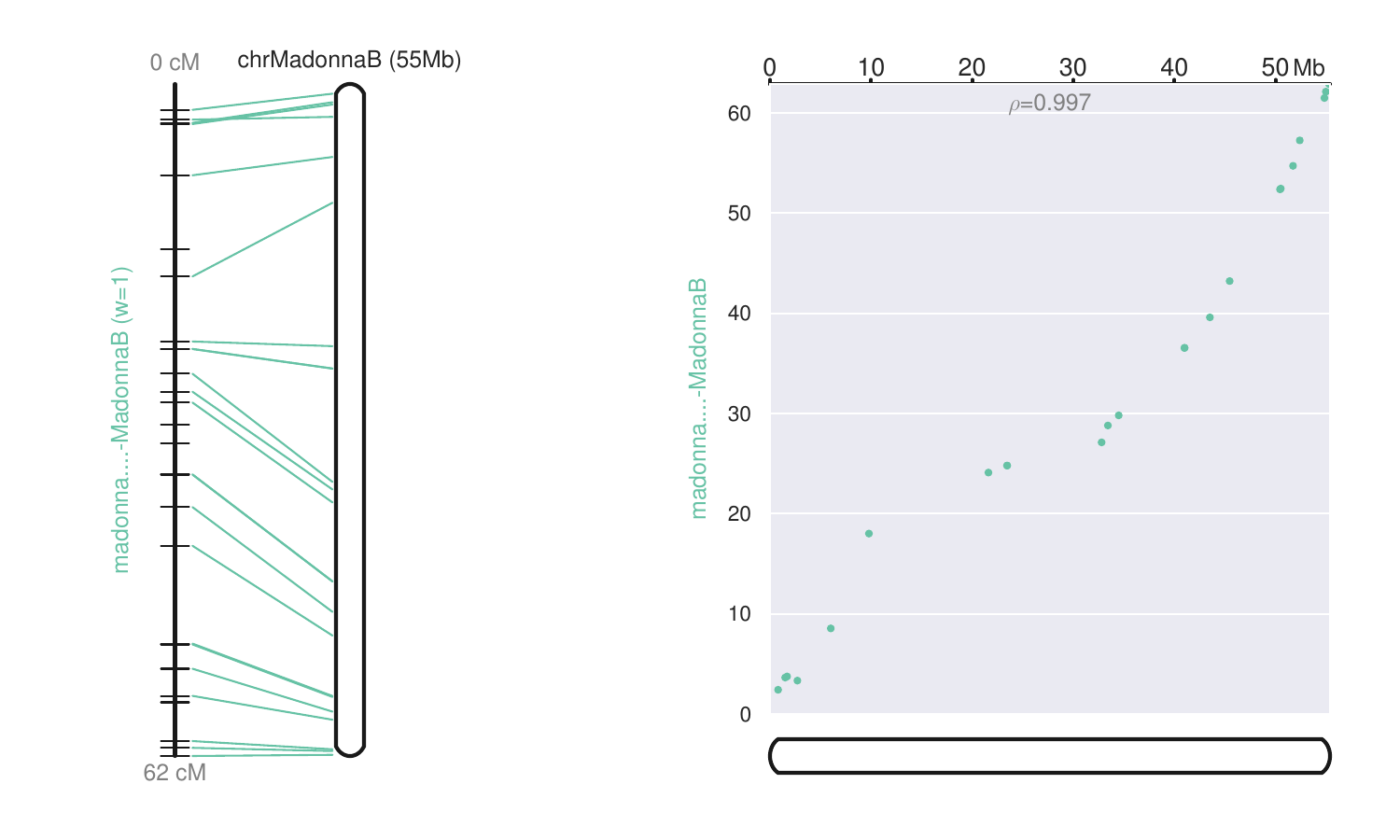

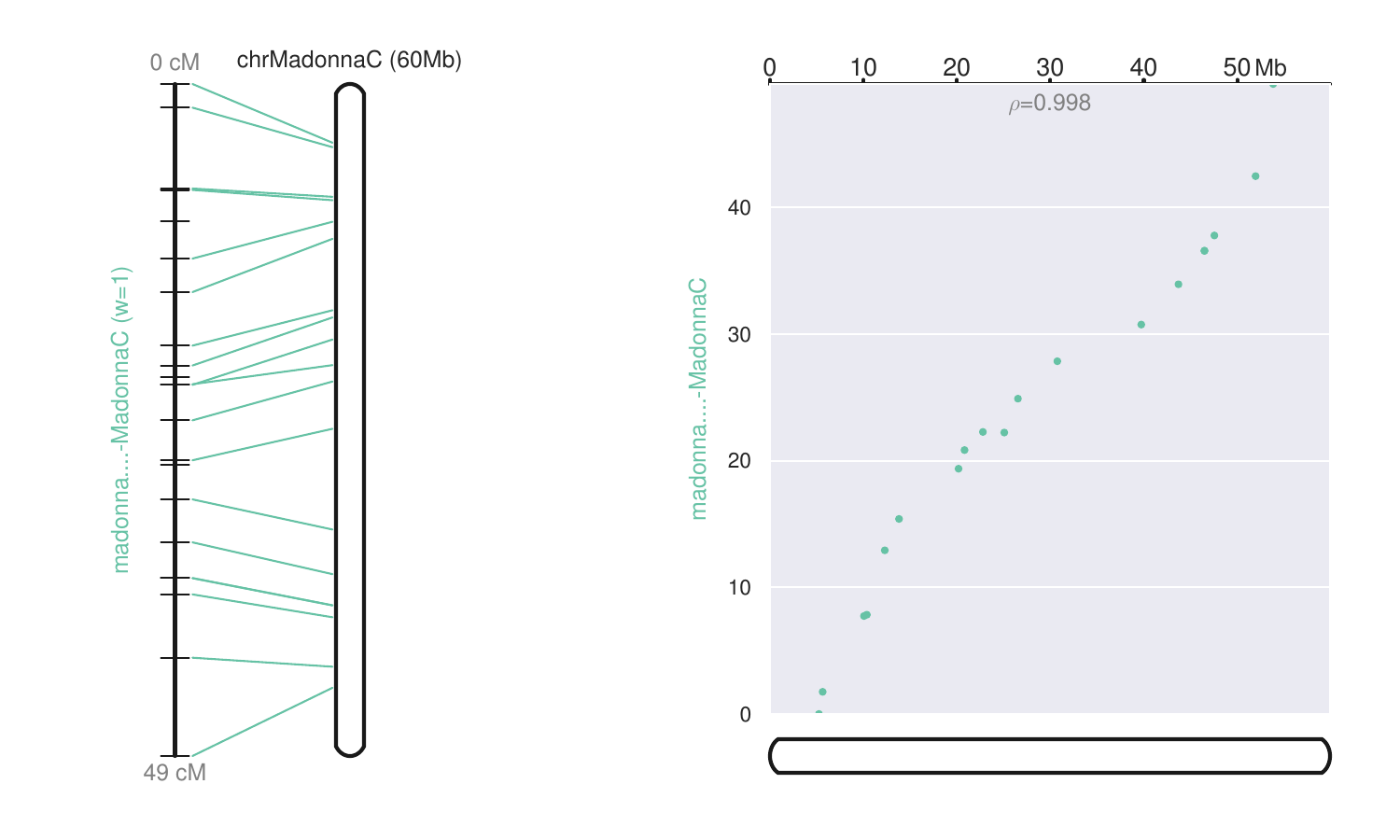

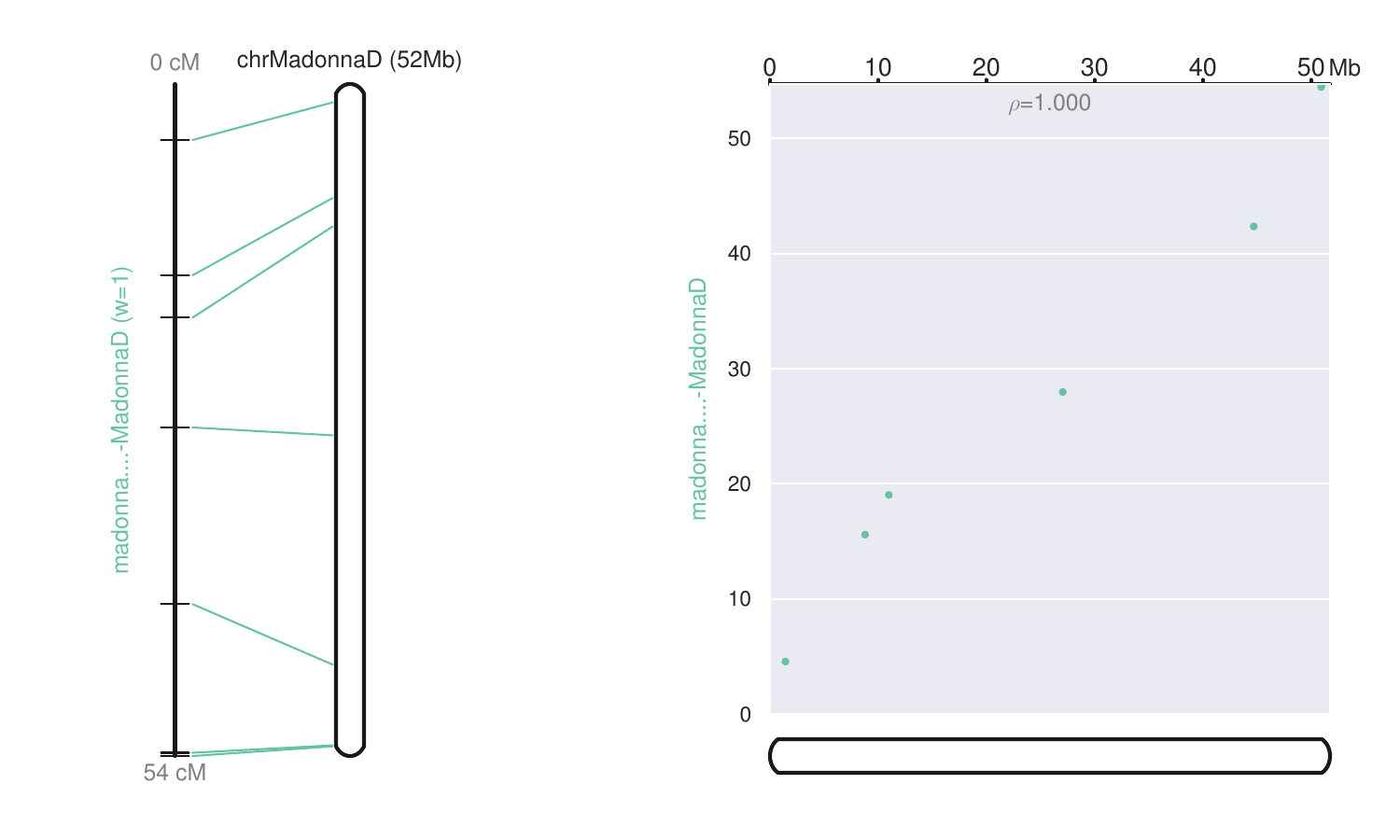

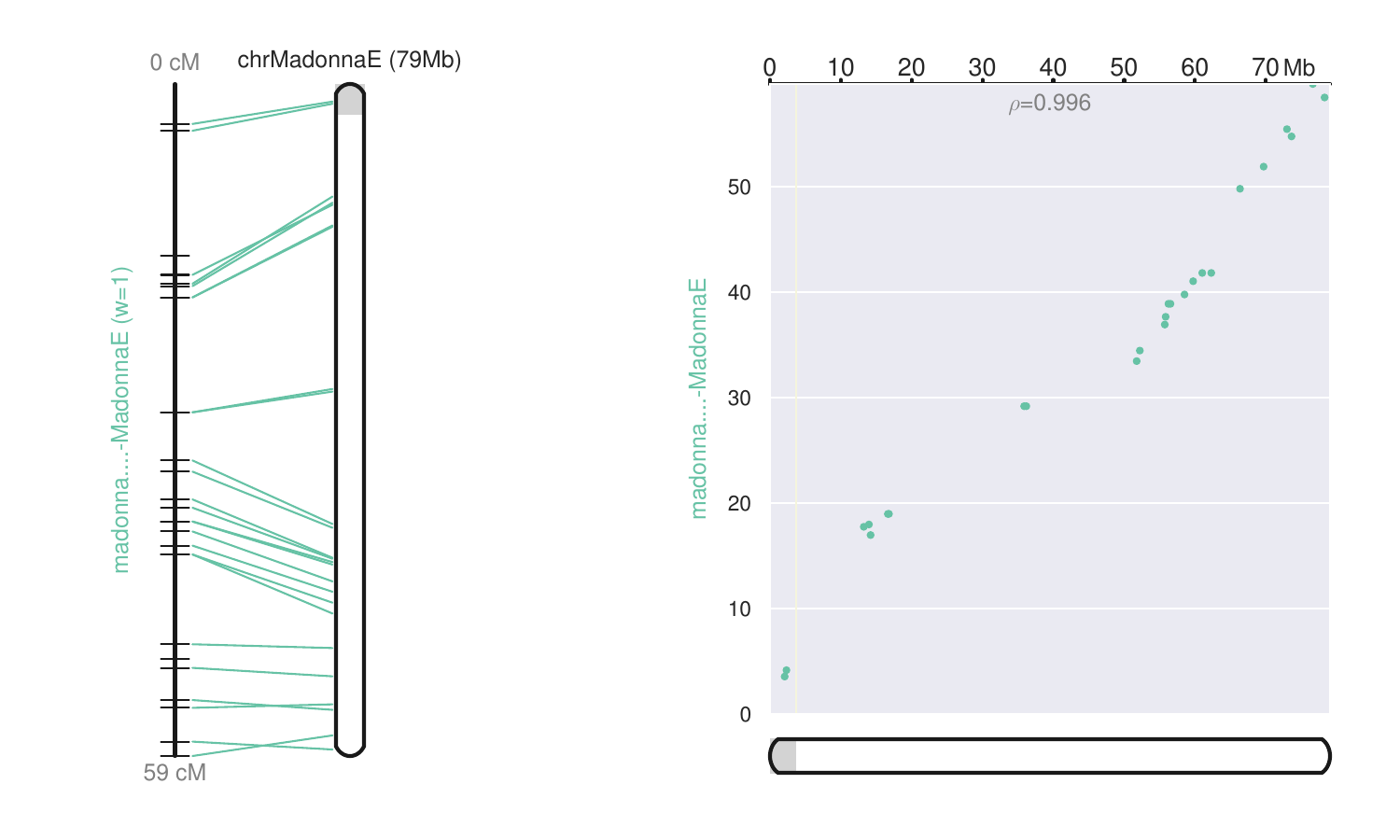

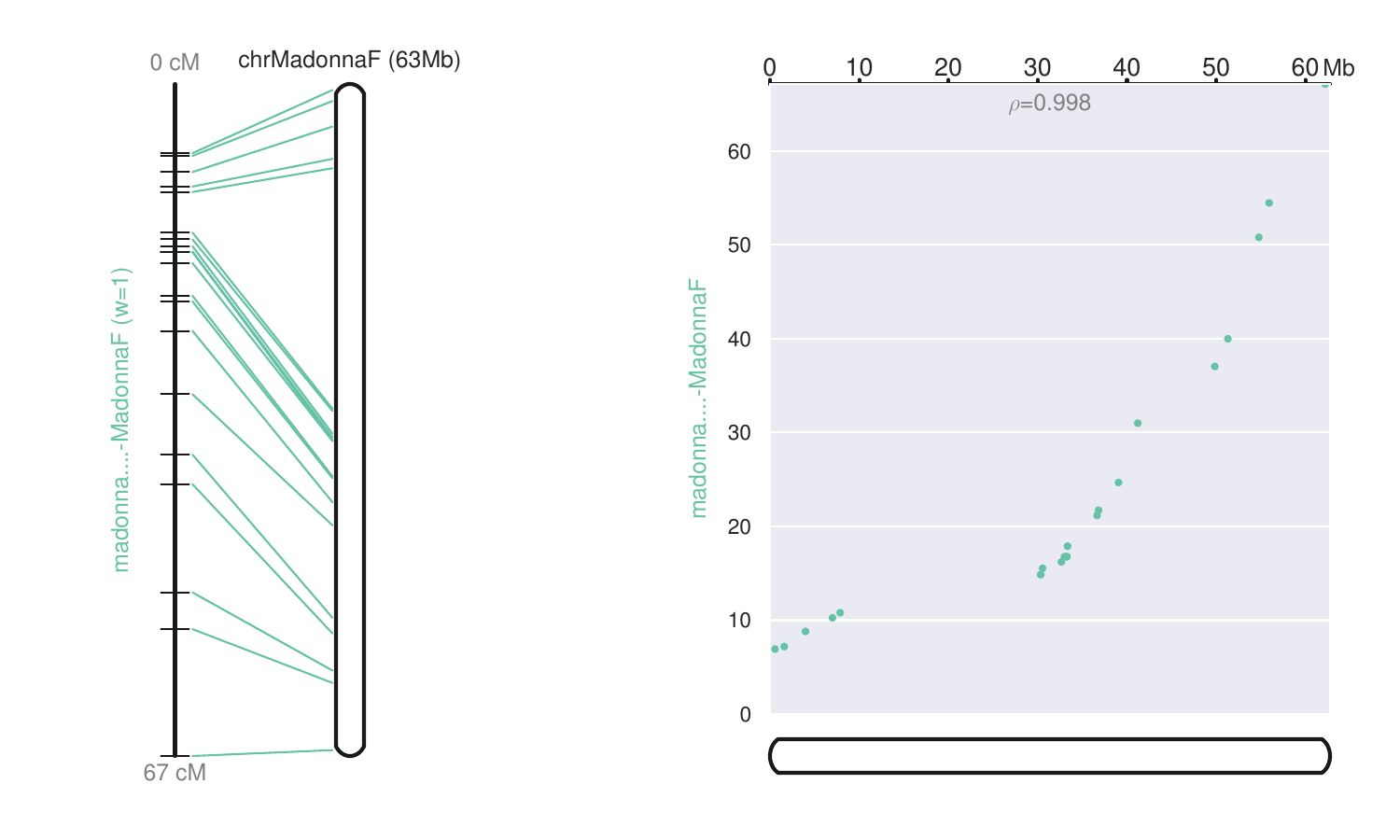

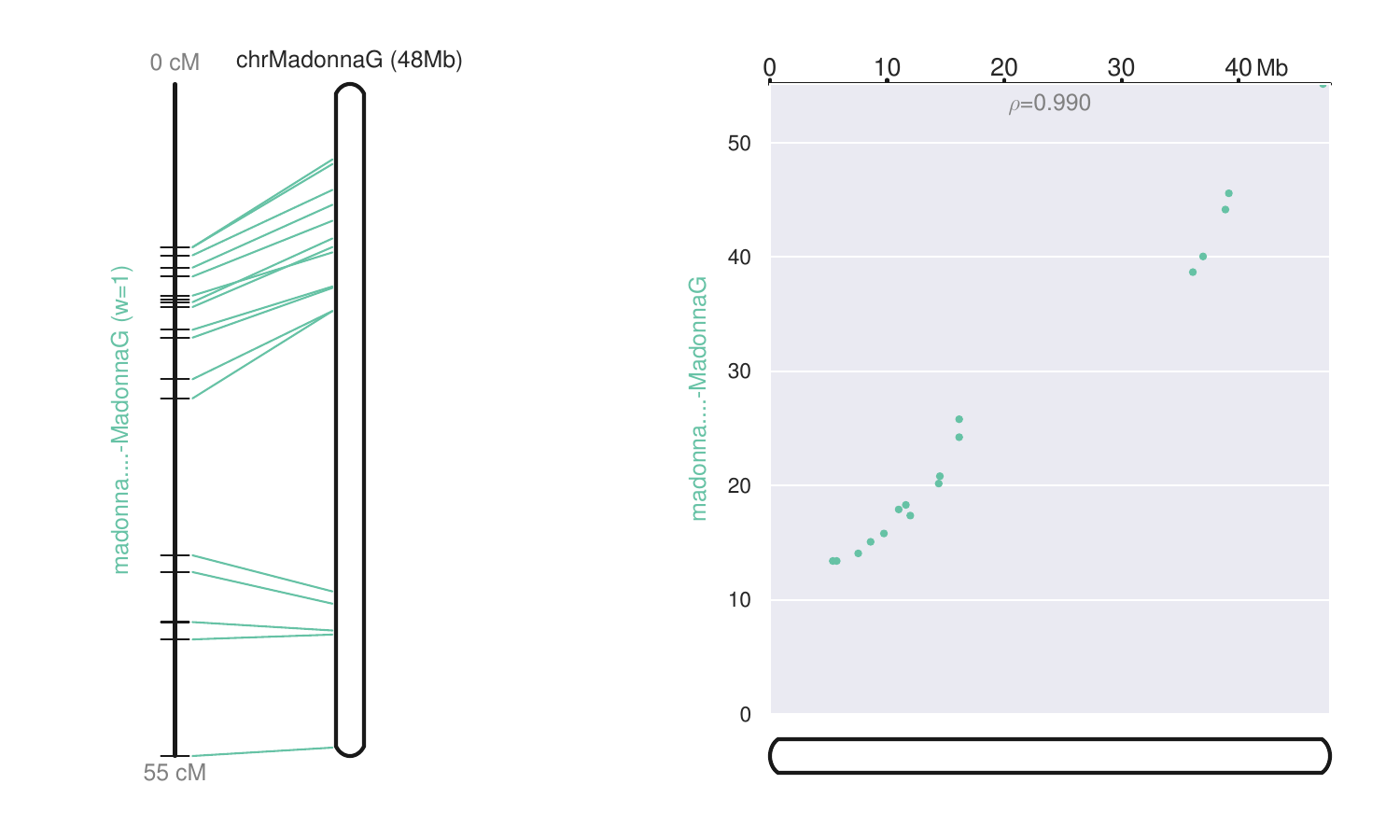

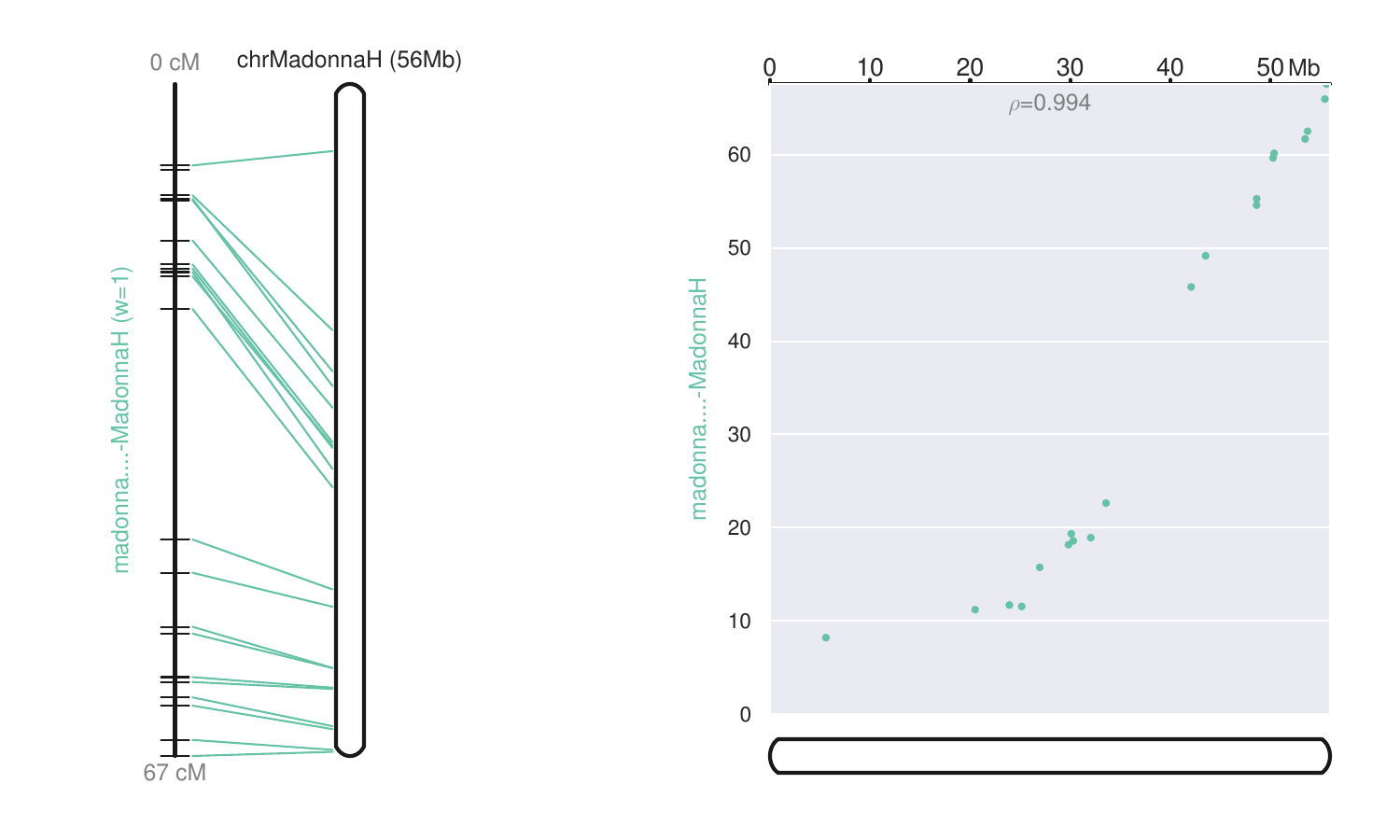

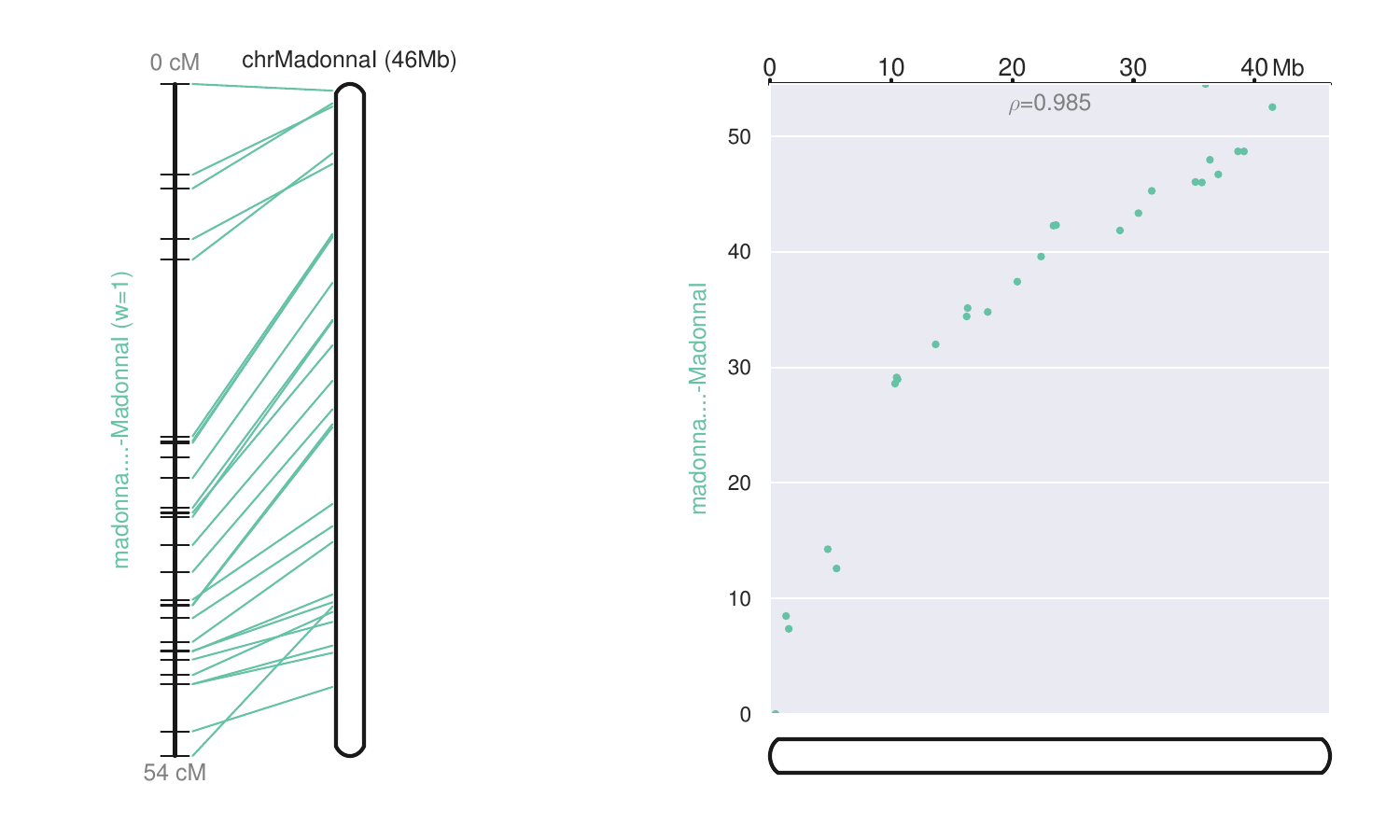

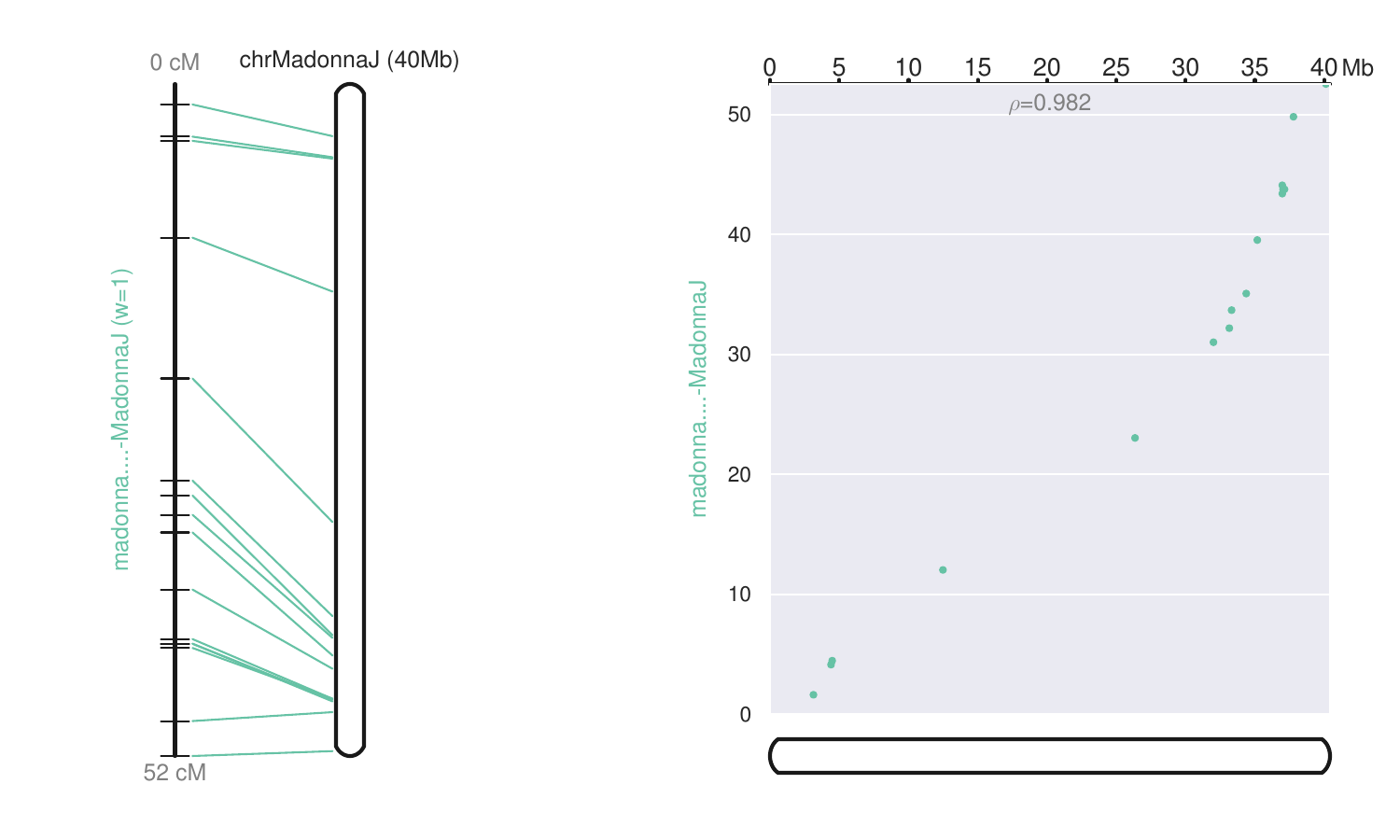

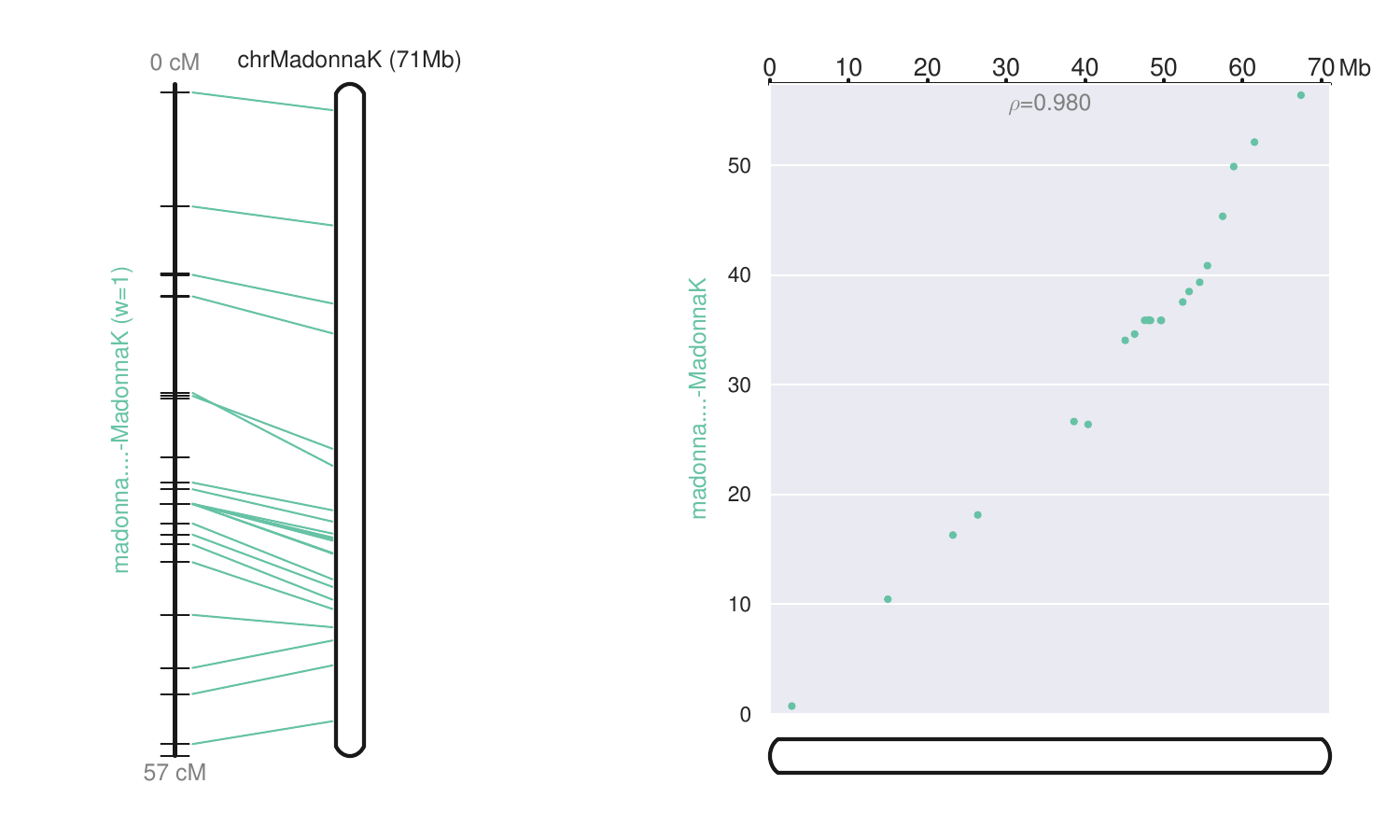

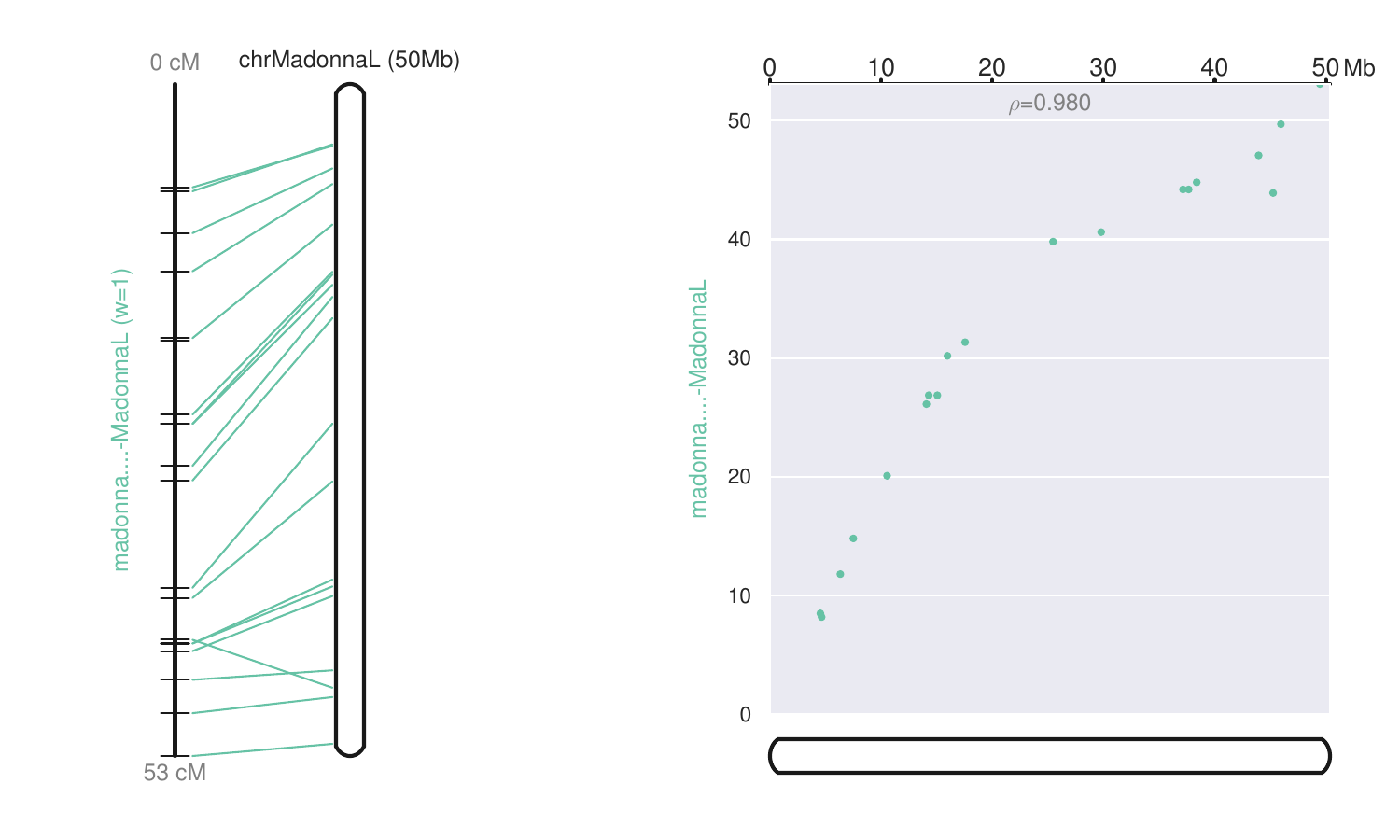


1. K-mer spectrum created with KAT (<https://kat.readthedocs.io/en/latest/installation.html>, [Bioinformatics.](https://www.ncbi.nlm.nih.gov/pmc/articles/PMC5408915/) 2017 Feb 15; 33(4): 574–576.). Kmers found in the reads are compared with the kmers of the assembly.


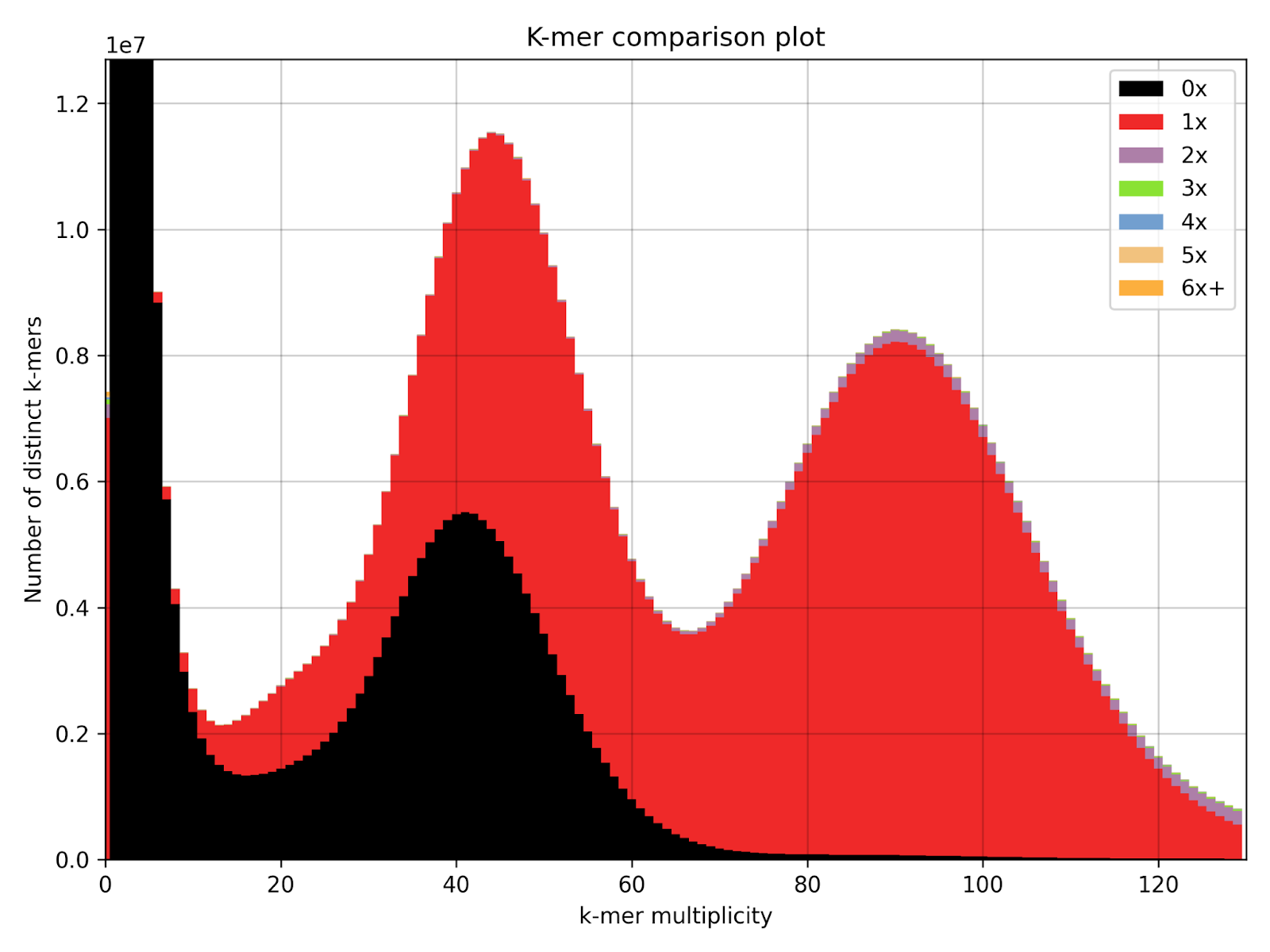


1. The summary of the BUSCO assessment.


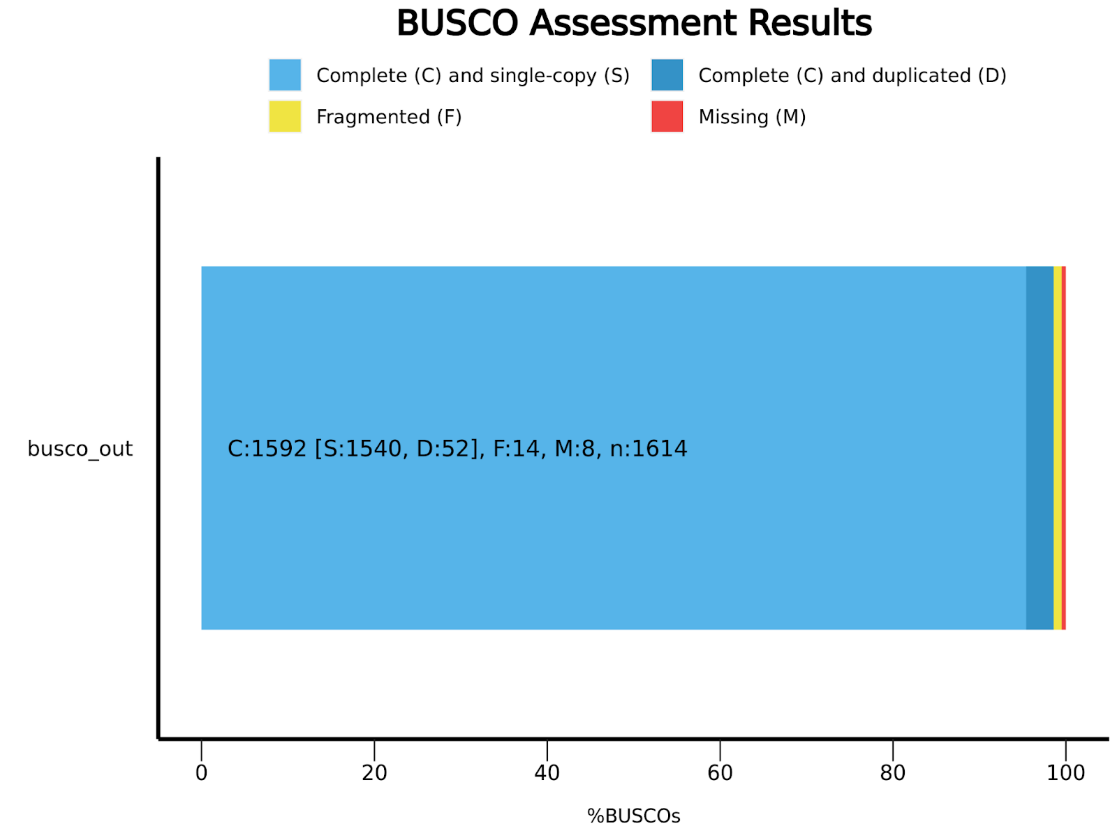

Supplement: Supplementary file 1 — Supplementary Material 1 [file 12863_2024_1245_MOESM1_ESM.docx]
